# Maternal Exposure to Cadmium and Manganese Impairs Reproduction and Progeny Fitness in the Sea Urchin *Paracentrotus lividus*

**DOI:** 10.1371/journal.pone.0131815

**Published:** 2015-06-30

**Authors:** Oriana Migliaccio, Immacolata Castellano, Paola Cirino, Giovanna Romano, Anna Palumbo

**Affiliations:** 1 Department of Biology and Evolution of Marine Organisms, Stazione Zoologica Anton Dohrn, Villa Comunale, Naples, Italy; 2 Marine Resources for Research Service, Stazione Zoologica Anton Dohrn, Villa Comunale, Naples, Italy; 3 Department of Integrative Marine Ecology, Stazione Zoologica Anton Dohrn, Villa Comunale, Naples, Italy; Laboratoire Arago, FRANCE

## Abstract

Metal contamination represents one of the major sources of pollution in marine environments. In this study we investigated the short-term effects of ecologically relevant cadmium and manganese concentrations (10^-6^ and 3.6 x 10^-5^ M, respectively) on females of the sea urchin *Paracentrotus lividus* and their progeny, reared in the absence or presence of the metal. Cadmium is a well-known heavy metal, whereas manganese represents a potential emerging contaminant, resulting from an increased production of manganese-containing compounds. The effects of these agents were examined on both *P*. *lividus* adults and their offspring following reproductive state, morphology of embryos, nitric oxide (NO) production and differential gene expression. Here, we demonstrated that both metals differentially impaired the fertilization processes of the treated female sea urchins, causing modifications in the reproductive state and also affecting NO production in the ovaries. A detailed analysis of the progeny showed a high percentage of abnormal embryos, associated to an increase in the endogenous NO levels and variations in the transcriptional expression of several genes involved in stress response, skeletogenesis, detoxification, multi drug efflux processes and NO production. Moreover, we found significant differences in the progeny from females exposed to metals and reared in metal-containing sea water compared to embryos reared in non-contaminated sea water. Overall, these results greatly expanded previous studies on the toxic effects of metals on *P*. *lividus* and provided new insights into the molecular events induced in the progeny of sea urchins exposed to metals.

## Introduction

Metals have been considered highly toxic pollutants and their presence in the environment has been increased in the last decades due to anthropogenic activities [1]. Aquatic ecosystems can be exposed to a great variety of metals whose chemical forms and concentrations are determined by different processes [2]. They can be distinguished in essential metals, which are required to support biological activities, and non-essential metals with unknown biological functions [3]. Some metals, including cadmium, have been extensively studied for many years [4] and many features have been identified such as the environmental persistence, the capacity of long-range transport, the bio-magnification along the food chain and bio-accumulation in animal tissues and the potential impact on humans and environmental health [5]. Other metals, such as manganese, essential in low amount in the organisms but toxic at high concentrations [6, 7], have only recently begun to be explored as emergent factors in the environmental contamination for the increasing production of metal-containing compounds. Both metals also differ in many aspects. Cadmium is one of the most widely distributed and dangerous pollutants for marine organisms [8–10]. Its concentration in the sea ranges from 10^−3^ to 2 μg/L depending on different factors, such as seawater latitudes [11, 12], depth [13] and pollution of different sites [14–16]. In some particular cases due to urbanization and industrialization, higher levels (0.2–72 mg/L) have been reported [9, 17]. Manganese, on the other hand, is a naturally occurring metal, toxic only at high levels [18–20]. Manganese concentration in marine environments is governed by pH, oxygen concentration and redox conditions. In natural sea water it ranges from 10 to 10,000 μg/L [21] but during hypoxia can reach values up to 22 mg/L [22, 23]. Toxic effects of metals have been investigated using different marine model systems and performing various biological assays/tests. The sea urchin *Paracentrotus lividus*, a key species in the Mediterranean sea, provides a unique and suitable tool for the evaluation of metal toxicity. The easy gamete preparation, the embryo transparency useful to detect several kinds of malformations, the relative synchrony and rapidity of development and the embryos/larvae sensitivity make the planktonic life stages suitable for embryo-toxicity tests [24, 25] and monitoring or risk assessment programs [15]. Moreover, *P*. *lividus* life is influenced by human activities, especially in the coastal zones [26]. These characteristics, together with its world-wide distribution, abundance and sedentary habits, prompted also the use of adult sea urchins as biological–biochemical indicators of local pollution [25, 27, 28].

The toxic effects of cadmium and manganese on sea urchin developing embryos have been extensively investigated [6, 7, 9, 29–33]. Recently, we have demonstrated that the physiological messenger nitric oxide (NO), produced by NO synthase (NOS) trough oxidation of L-arginine, mediates the stress response induced by environmentally relevant concentrations of cadmium and manganese in *P*. *lividus* developing embryos. Moreover, by using pharmacological and molecular approaches we found that the transcriptional expression of some metal-induced genes involved in stress response, skeletogenesis, detoxification and multi-drug efflux was directly or indirectly regulated by NO [10]. Interestingly, NO is also involved in the response of *P*. *lividus* embryos to the toxic diatom-derived aldehyde decadienal [34]. On the contrary, only few studies have been performed to understand the effects of metals on adult sea urchins and their offspring. The progeny of *Strongylocentrotus intermedius* sea urchin, collected in the coastal zone of Amur Bay, characterized by an extensive pollution, showed a delayed development together with a large number of anomalies [8, 35, 36]. Similar results were also obtained in other studies on sea urchins collected from other polluted habitats (e.g. Naidenko) [37]. In *Sphaerechinus granularis* from the Bay of Brest, a large number of blocked and delayed embryos were observed, due to the presence of high levels of heavy metals in sea urchin gonads [38].

In this study we investigated the effects of cadmium and manganese, at concentrations mimicking polluted sea water, on female sea urchins *P*. *lividus* exposed for 2 and 9 days and on their offspring. Here, we show that both metals differentially impaired the fertilization process causing modifications in the reproductive state. Moreover, we found an abnormal development of the offspring of exposed females associated to a rise in NO levels in gonads and changes in the transcription of several genes involved in stress response, skeletogenesis, detoxification, multi drug efflux processes and NO production.

## Materials and Methods

### Ethics statement

*Paracentrotus lividus* (Lamarck) sea urchins were collected in the Gulf of Naples, near Castel dell’Ovo (40° 49’ 41” latitude, 14° 14’ 48” longitude), from a location that is not privately-owned nor protected in any way, according to the authorization of Marina Mercantile (DPR 1639/68, 09/19/1980, confirmed by D. Lgs. 9/01/2012 n.4). The field studies did not involve endangered or protected species. All animal procedures were in compliance with the guidelines of the European Union (directive 2010/63 and following D. Lgs. 4/03/2014 n.26).

### Adult acclimation, treatments and gonads collection

Sea urchins were collected during the breeding season by SCUBA divers from the Gulf of Naples, transported in an insulated box to the laboratory within 1 h after collection and maintained in tanks with circulating sea water (1 animal/5 L). The animals were acclimated for a minimum of 10 days and kept in a controlled temperature chamber at 18±2°C with 12:12 light:dark cycle. Every 3 days animals were fed *ad libitum* by using fresh macroalgae (*Ulva* sp). Feeding was interrupted 2 days before experimental sampling. Rare spontaneous spawning and mortalities were observed during the acclimation period. Females (4.1 ± 0.98 cm), identified by Dr. Davide Caramiello from the service Marine Resources for Research of the Stazione Zoologica through observation at the stereomicroscope, were selected, weighed and transferred in experimental tanks. Each tank contained a group of 6 to 8 animals, as reported in figure legends, and 5 L of sea water per animal. Females were exposed to sea water containing cadmium 10^−6^ M (0.183 mg/L) or manganese 3.6x10^-5^ M (5.83 mg/L), prepared by stock solutions of 10^−4^ M cadmium (cadmium chloride, Sigma-Aldrich, Milan, Italy) and 31.2x10^-4^ M manganese (manganese chloride tetrahydrate, Sigma-Aldrich, Milan, Italy). Metal treatments were performed for 2 and 9 days. Control experiments were carried out by keeping the animals in sea water, in the tanks, as reported above, without addition of metals. The experimental tanks were artificially aerated and kept at 18±2°C with 12:12 light:dark cycle. Twice a week animals were fed with rations meal of *Ulva* sp. and 30% of the water was removed and replaced with new sea water containing the metal at the experimental concentration. All experiments were performed at least in triplicate. After 2 and 9 days, females were collected and weighed. Ovaries were removed, weighed, washed with PBS, frozen in liquid nitrogen and kept at -80°C until analysis. Moreover, eggs from single animals were collected and fertilized as described below.

### Determination of gonadosomatic index, spawning and fertilization success

The gonadosomatic index (GSI) of the females was calculated as the ratio of the gonad mass to the whole-body wet mass (%). To induce gamete ejection, sea urchins were injected with 0.5 M KCl through the peribuccal membrane. Eggs from individual females were washed three times with 0.22 μm filtered sea water. Concentrated sperm was collected dry, mixing samples from at least three different males and keeping undiluted at + 4°C. 10 μL of sperm mix was diluted in 10 mL sea water just before fertilization and an aliquot (100 μL) of this solution was added to 100 mL of egg suspension. Sperm to egg ratio was 100:1 for both controls and treated embryos. The fertilization success was approximately 90%. The spawning was determined as the ratio between the number of spawning females and the total number of females (%). The fertilization success was calculated as the ratio of the fertilized eggs observed at the first division (1h) respect to the number of total eggs (%).

### Gamete collection, embryo culture, treatments and morphological analysis

Animals were sacrified and gonads were gently washed to allow egg release. The fertilization procedure was as described by Migliaccio et al. [10] with slight modifications. Briefly, eggs were collected from treated and control females and kept in sea water. The sperm from 3 control males was pooled and maintained dried in an eppendorf in cold conditions (+ 4°C) until fertilization. Diluted sperm (1:1000) was added to 100 mL of egg suspension (15000 eggs). Sperm to egg ratio were 100:1 for both controls and treated embryos. The mixture was carefully stirred to allow fertilization to take place. Five min after fertilization, eggs from treated animals were divided in two different groups. The eggs from the first group were reared in normal sea water whereas the eggs of the second group were reared in metal-containing sea water (cadmium 10^−6^ M or manganese 3.6 x 10^−5^ M). As control, eggs from females, maintained in tanks without metals for 2 and 9 days, were fertilized in sea water with a fertilization success approximately of 90%. Fertilized eggs were allowed to develop in a controlled temperature chamber at 18±2°C and 12:12 light:dark cycle. The development was followed by inverted microscope (Zeiss Axiovert 135 TV) until the pluteus stage, approximately 48 hours post fertilization (hpf). Morphological observations were performed on plutei fixed in 4% formalin. Embryos were considered normal if they reached the pluteus stage of development, exhibited good body symmetry, showed fully developed skeletal rods and displayed a well differentiated gut. All the morphologies that did not satisfy the above-mentioned criteria were grouped and referred to as abnormal [16, 39, 40].

### NO determination

The endogenous NO levels were measured by monitoring nitrite formation by Griess reaction [10, 41]. Collected ovaries, washed in PBS and frozen in liquid nitrogen, were homogenized in 20 volumes of PBS and centrifuged at 25,000 x g for 20 min at + 4°C. The supernatants were analyzed for nitrite content. Sea urchin developing embryos were collected at different developmental stages (early blastula, swimming blastula, prism and pluteus stages) by centrifugation at 1800 g for 10 min in a swing out rotor at + 4°C. The pellet was washed with PBS, frozen in liquid nitrogen and kept at -80°C until use. Samples were homogenized in PBS (1:2 w/v) and centrifuged (12,000 g for 30 min at 4°C) and the supernatants were analyzed for nitrite content.

### RNA extraction and cDNA synthesis

Embryos at different stages of development (about 1500) were collected by centrifugation as described above. Total RNA was extracted from each developmental stage, namely: early blastula, swimming blastula, prism and pluteus, using RNAqueous-Microkit (Ambion) according to the manufacturer’s instructions. The amount of total RNA extracted was estimated by the absorbance at 260 nm and the purity by 260/280 and 260/230 nm ratios by Nanodrop (ND-1000 UV–Vis Spectrophotometer; NanoDrop Technologies). The integrity of RNA was also evaluated by agarose gel electrophoresis. Intact rRNA subunits (28S and 18S) were observed on the gel indicating minimal degradation of the RNA. For each sample, 600 ng of total RNA extracted was retrotranscribed with iScript cDNA Synthesis kit (Biorad), following the manufacturer’s instructions. cDNA was diluted 1:5 with H_2_O prior to use in Real Time qPCR experiments.

### Gene expression by real time qPCR

Real time qPCR experiments were performed on the offspring of sea urchin females exposed to cadmium and manganese for 2 and 9 days, with respect to the offspring of females reared in sea water in the absence of metals. The data from each cDNA sample were normalized using *Pl-Z12-1* as reference gene, because its level remained constant during development [10, 42]. The following genes were analyzed: *hsp70*, *hsp60*, *hsp56*, *sm30*, *sm50*, *p16*, *p19*, *msp130*, *bmp5-7*, *fg9/16/20*, *mt4*, *mt5*, *mt6*, *mt7*, *mt8*, *abc1a*, *abc4a*, *abc1b*, *abc8b* and *nos*. For all genes we used primers reported in Migliaccio et al. [10]. Diluted cDNA was used as a template in a reaction containing a final concentration of 0.3 μM for each primer and 1× FastStart SYBR Green master mix in a total volume of 10μL. PCR amplifications were performed in a ViiA7 Real Time PCR System (Applied Biosystems) thermal cycler using the following thermal profile: 95°C for 10 min, one cycle for cDNA denaturation; 95°C for 15 s and 60°C for 1 min, 40 cycles for amplification; 72°C for 5 min, one cycle for final elongation; one cycle for melting curve analysis (from 60°C to 95°C) to verify the presence of a single product. Each assay included a no-template control for each primer pair. To reduce intra-assay variability all Real Time qPCR reactions were carried out in triplicate. Moreover, at least 3 biological replicates were performed. Fluorescence was measured using ViiA7 Software (Applied Biosystems). The expression of each gene was analyzed and internally normalized against *Pl-Z12-1* using Relative Expression Software Tool software (REST) based on the method by Pfaffl et al. [43]. Relative expression ratios equal or greater than two fold were considered significant.

### Statistical analysis

Data are presented as means ± SD. Two-way ANOVA (*P* < 0.05) with Bonferroni post hoc test was used to analyze data. Statistics was performed with GraphPad Prism 4.0 for Windows (GraphPad Software, San Diego, CA, USA). For Real Time qPCR analysis, significance was tested using the “Pair Wise Fixed Reallocation Randomisation Test”, developed by REST software [43]. The number of experiments is reported in figure legends.

## Results

### Effects of cadmium and manganese on reproductive state and nitric oxide (NO) production in *P*. *lividus* females

**Fig 1:**
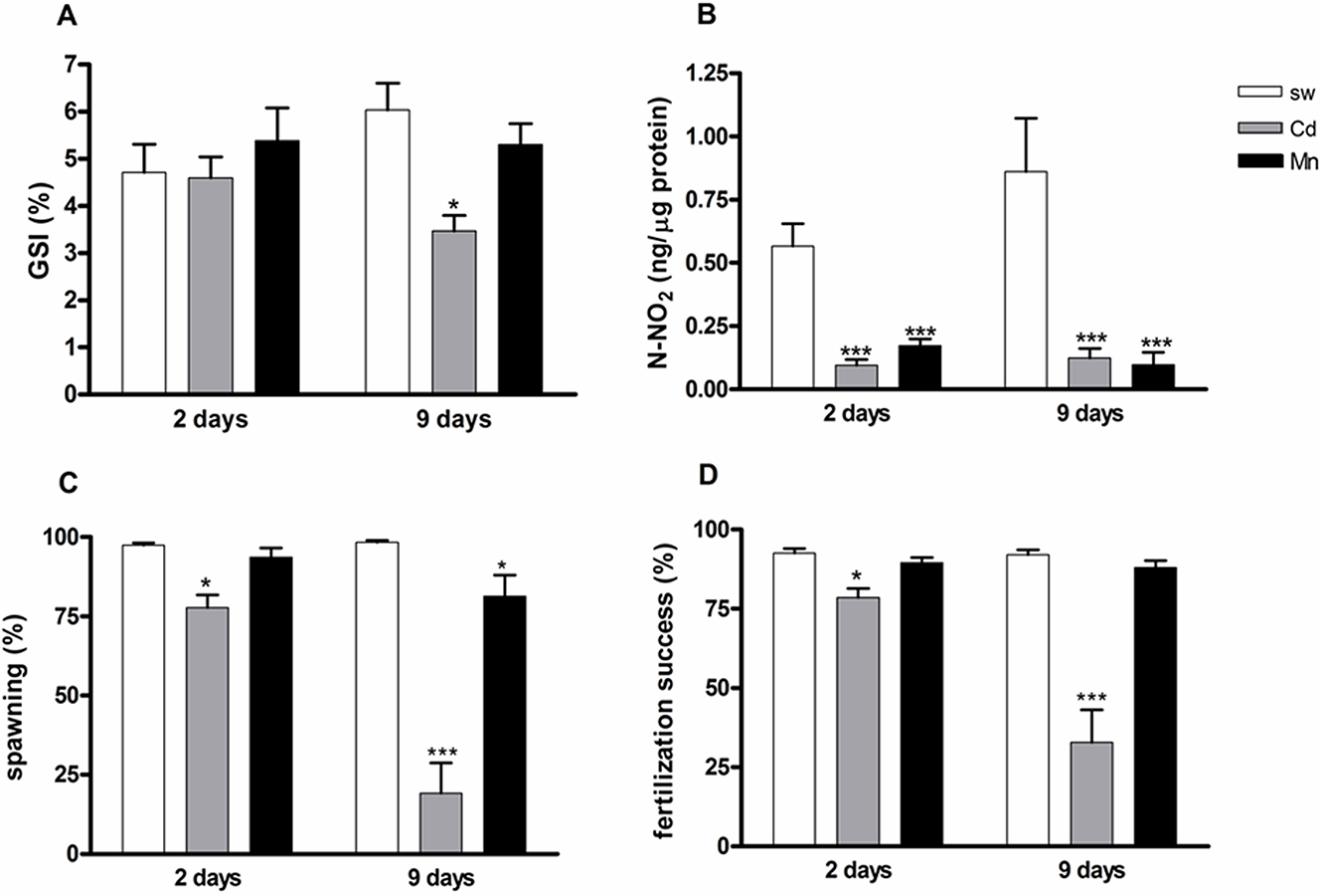
Reproductive state and nitric oxide (NO) production in *P*. *lividus* females exposed to cadmium and manganese. GSI (A), total NO concentration (B), spawning (C) and fertilization success (D) in females exposed to cadmium (Cd) 10^−6^ M and manganese (Mn) 3.6 x 10^−5^ M for 2 and 9 days. Significant differences compared to the controls (sw 2 and 9 days): *P<0.05, ***P<0.001. Two-way ANOVA, Bonferroni’s post test (P<0.05). N = 8.

Selected females were exposed for 2 and 9 days to cadmium 10^−6^ M and manganese 3.6 x 10^−5^ M. These metal concentrations were shown to be sublethal for developing *P*. *lividus* embryos, inducing a small percentage of abnormal plutei (28–29%) after treatment of fertilized eggs [10]. Then, the reproductive state was assessed by determination of gonadosomatic index (GSI), spawning and fertilization success. Moreover, NO content was measured in the ovaries as total nitrite by Griess assay. The exposure of sea urchin females to cadmium for 2 days caused a significant reduction in spawning (Fig 1C), fertilization success (Fig 1D) and NO production (Fig 1B), compared to the respective controls, females reared in sea water without metals. No effect was observed on GSI (Fig 1A). After 9 days, all parameters were affected by metal treatment. In particular, a significant reduction in GSI (Fig 1A), NO production (Fig 1B), spawning (Fig 1C) and fertilization success (Fig 1D) was recorded in cadmium-exposed sea urchins compared to the respective controls. Manganese exposure caused a decrease in NO levels at both experimental times (Fig 1B) and only a reduction in spawning after 9 days of treatment (Fig 1C).

### Effects of cadmium and manganese on offspring of exposed *P*. *lividus* females

To investigate the effects of cadmium and manganese on the progeny of exposed sea urchins, the development of the offspring from females exposed to cadmium 10^−6^ M or manganese 3.6 x 10^−5^ M for 2 and 9 days and reared in the absence or in the presence of the metal was followed by morphological analysis, NO production and gene expression. We considered as control the offspring of females kept during the whole experimental period in sea water without addition of metals. Morphological analysis was performed at the pluteus stage, whereas NO production and gene expression were examined at different developmental stages: early blastula, swimming blastula, prism and pluteus.

#### Morphological analysis

**Fig 2:**
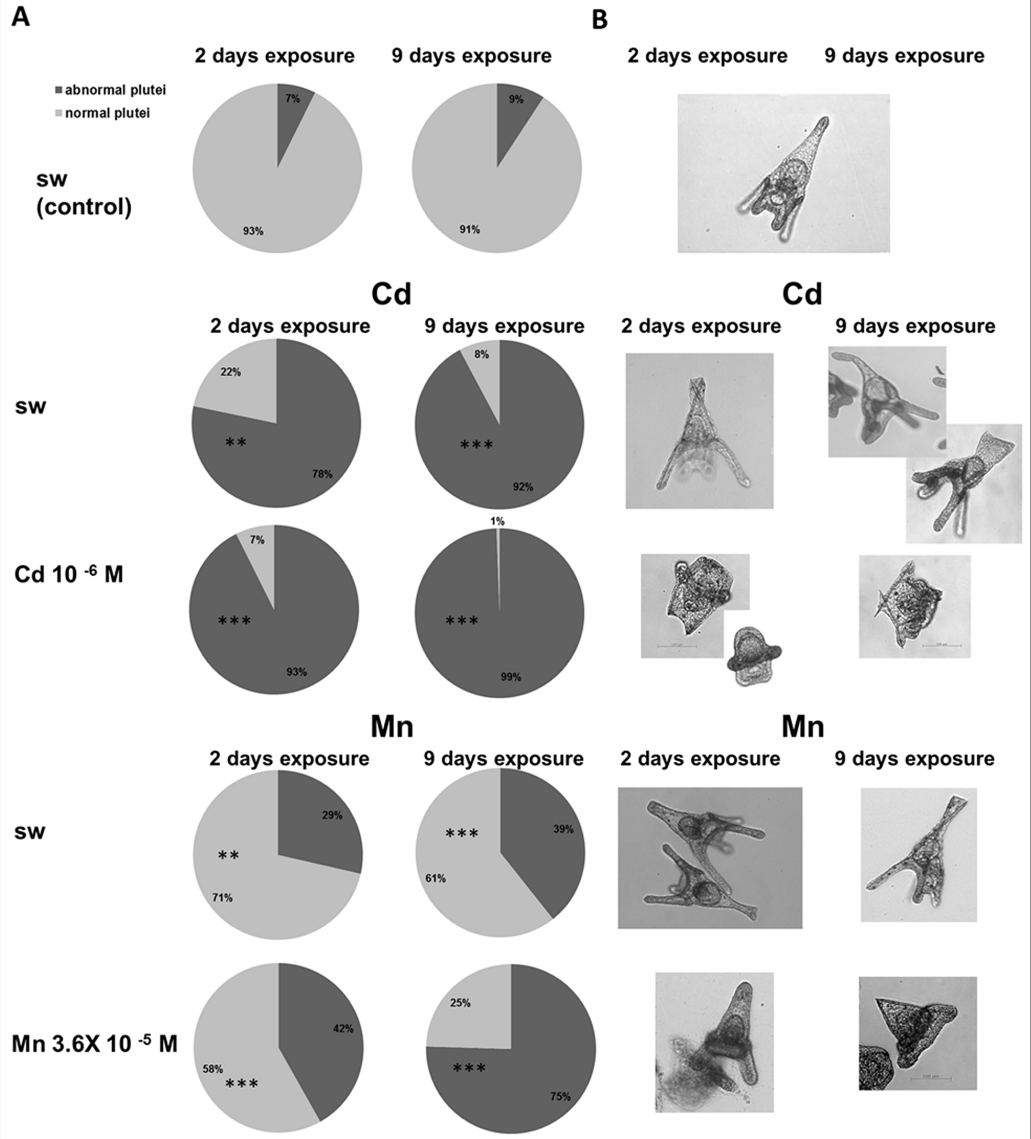
Morphological analysis of the progeny of *P*. *lividus* females exposed to cadmium and manganese. Females were treated for 2 and 9 days with cadmium (Cd) 10^−6^ M and manganese (Mn) 3.6 x 10^−5^ M, as described in Materials and Methods. Offspring was reared in sea water (sw) or in the presence of the metal. The development was monitored after 48 hpf. A. Percentage of normal and abnormal plutei; B. Representative pictures of the main abnormalities (bar = 100 μm). Significant differences compared to the control **P<0.01, ***P<0.001; Two-way ANOVA (*P*<0.05), with Bonferroni’s Post Test. Light grey: normal plutei; dark grey: abnormal plutei. N = 8.

For morphological analysis, we considered as normal plutei cone-shaped larvae with four fully developed arms and complete skeletal rods, whereas larvae with defects in arm and skeleton elongation and developmentally delayed were named abnormal, as previously described [10]. The exposure of females to both cadmium and manganese resulted in an increase in the percentage of abnormal plutei in the progeny (Fig 2A). In particular, the offspring of females exposed to cadmium 10^−6^ M reared in sea water showed 78±4.2% and 92±3.7% of abnormal plutei after 2 and 9 days respectively (Fig 2A), compared to the respective controls (7±1.2 and 9±0.73%). Defects were found in both arms and apex. The arms appeared often malformed or absent, whereas skeletal rods of the apex were folded, crossed or separated (Fig 2B). An increase in the number and type of abnormalities was found in the offspring of exposed females reared in the presence of cadmium with a percentage of abnormal plutei of 93±7.23 and 99±0.54% after 2 and 9 days, respectively, compared to controls in the absence of cadmium (Fig 2A). Larvae appeared severely affected by metal treatment with high percentage of gastrula and prism-like stages and arrested embryos (Fig 2B). The exposure of females to manganese 3.6 x 10^−5^ M also caused abnormality in the offspring. In details, the embryos generated from those females and reared in sea water reached values of abnormalities of 29±3.23 and 39±4.65 after 2 and 9 days, respectively (Fig 2A), compared to the respective controls, offspring of females kept during the whole experimental period in sea water without metal. The abnormalities regarded especially the arms which appeared shorter than normal or malformed and the skeletal rods of the apex which were crossed or separated (Fig 2B). As in the case of cadmium treatment, an increase in the percentage and type of abnormalities was found in embryos reared in the presence of manganese which reached the values of 42±2.12 and 75±1.9% after 2 and 9 days, respectively, compared to controls. Larvae appeared much smaller in size and defects were found in both arms and apex (Fig 2B).

#### NO production

**Fig 3:**
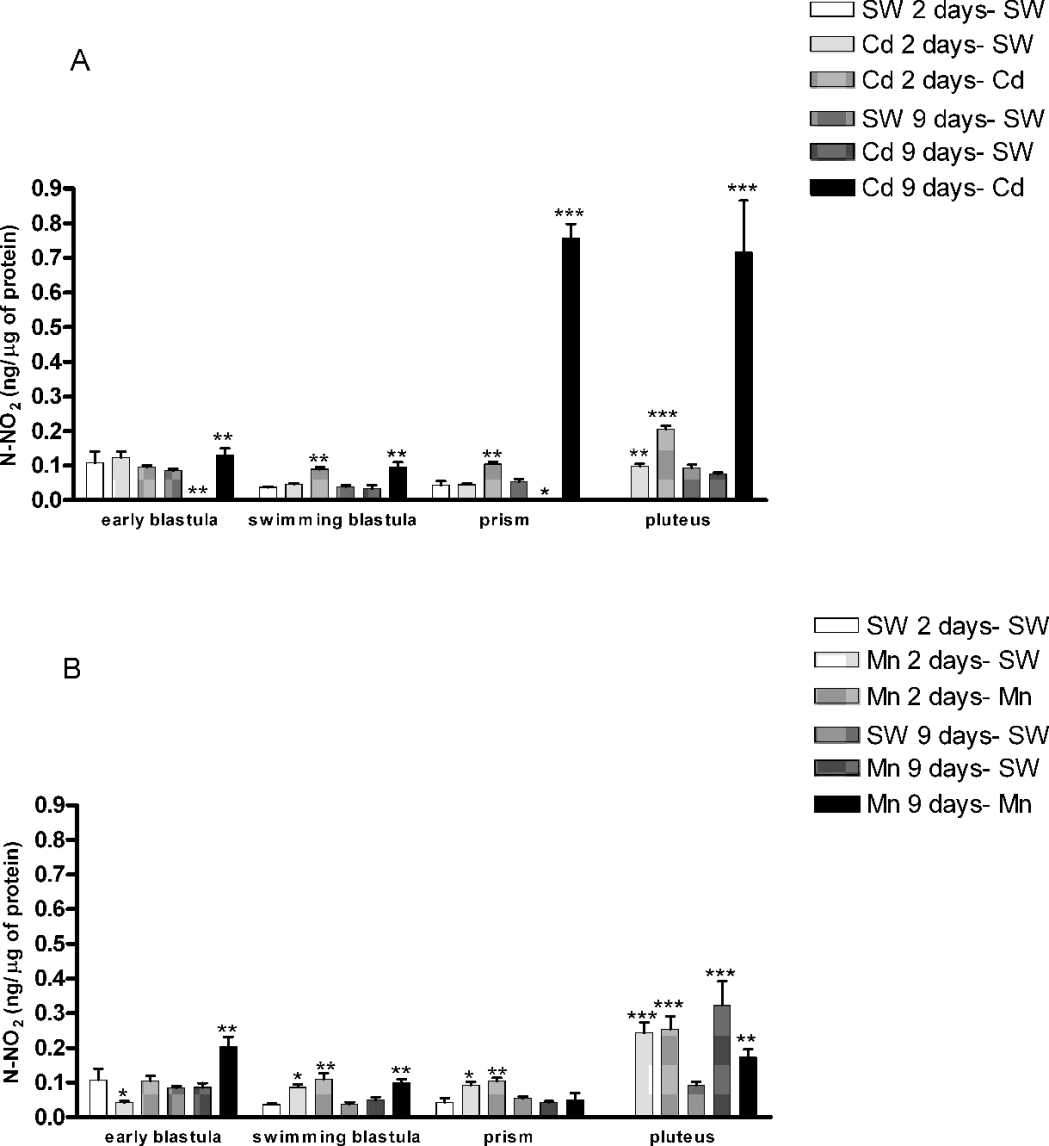
Total NO concentration in the progeny of *P*. *lividus* females exposed to cadmium and manganese. Different developmental stages of the offspring of females treated with cadmium (Cd) 10^−6^ M (A) or manganese (Mn) 3.6 x 10^−5^ M (B) for 2 and 9 days and reared in sea water (SW) or in SW containing metals were examined for nitrite content, as reported in Material and Methods. Significant differences compared to the respective control (SW 2 and 9 days) *P<0.5, **P<0.01, ***P<0.001. Two-way ANOVA, Bonferroni’s post test (*P*<0.05). N = 6.

We examined the different developmental stages of the offspring of females exposed to cadmium and manganese for NO content, measured as total nitrite. As shown in Fig 3, the offspring of females treated for 2 days with cadmium 10^−6^ M and reared in normal sea water showed a significant increase in total nitrite with respect to the control, only at the pluteus stage, whereas embryos reared in the presence of cadmium showed increased NO production at the swimming blastula, prism and pluteus stages, compared to the respective controls. After 9 days of cadmium exposure, offspring reared in sea water without metal, showed a decreased NO production at the early blastula and prism stages, compared to the respective controls, whereas embryos reared in the presence of cadmium exhibited an increased NO production at all developmental stages (Fig 3A), compared to the controls. Also manganese affected the production of NO. In fact, the offspring of females exposed for 2 days to manganese, reared in sea water, showed a reduction in total nitrite at the early blastula stage, whereas at all other developmental stages a significant increase was revealed compared to the controls. Also offspring reared in manganese-containing sea water showed an increased NO production at the swimming blastula, prism and pluteus stages, with respect to the controls (Fig 3B). The offspring reared for 9 days in normal sea water showed a significant increase in NO production at the pluteus stage. On the contrary, embryos reared in sea water containing manganese increased total nitrite at almost all developmental stages, except at the prism stage, compared to the respective controls.

#### Gene expression

**Fig 4:**
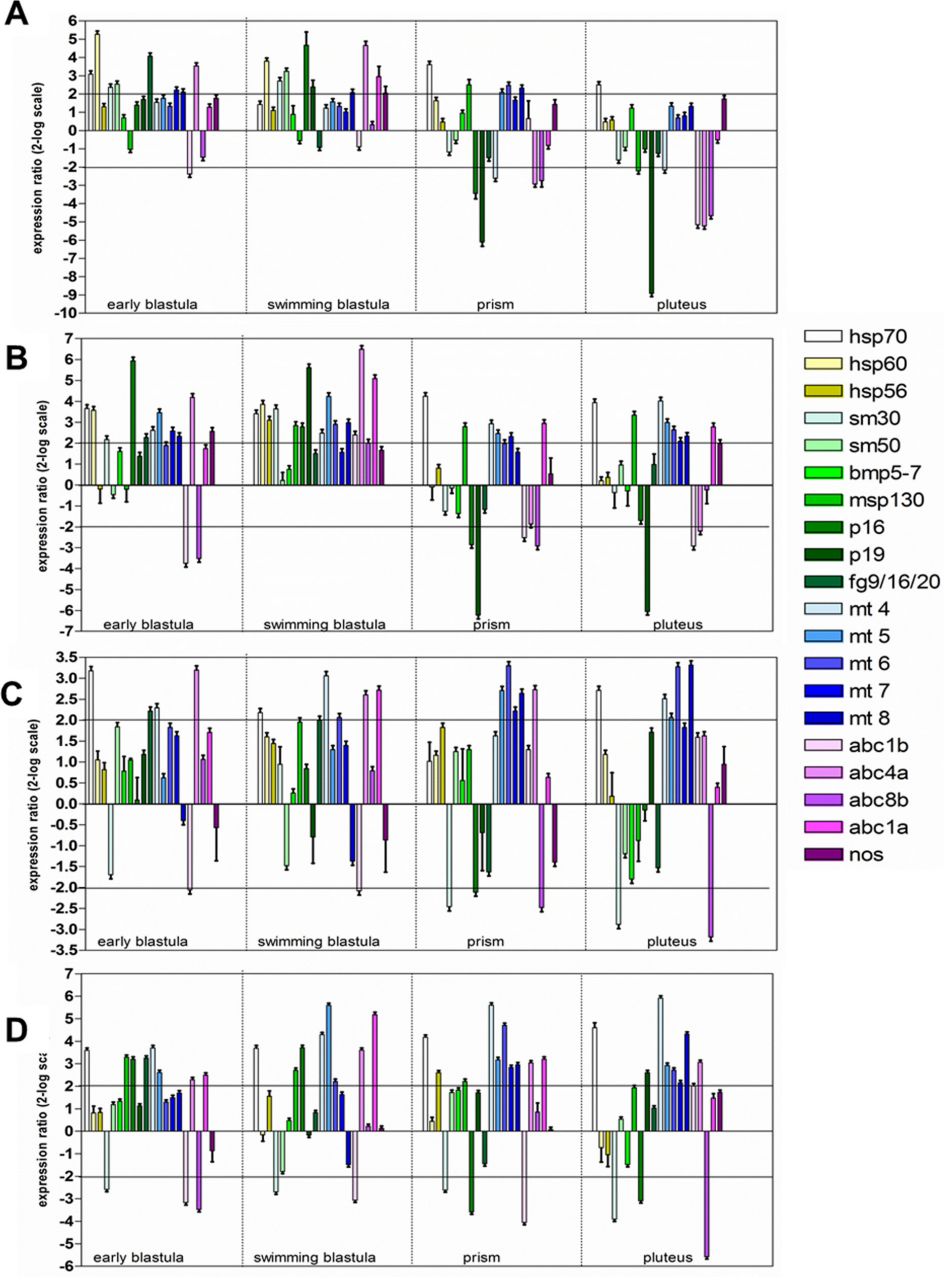
Gene expression analysis in the progeny of *P*. *lividus* females exposed to cadmium 10^−6^ M for 2 days (A,B) and 9 days (C,D). The embryos were reared in sea water (A,C) and in cadmium-containing sea water (B,D). Data are reported as a fold difference in the expression levels of the analyzed genes, compared to controls (mean ± SD), offspring of females kept during the whole experimental period in sea water without addition of metal. Fold differences equal or greater than ± 2 (see horizontal guidelines at values of 2 and—2) were considered significant. Experiments were repeated at least on 3 biological replicates.

**Fig 5:**
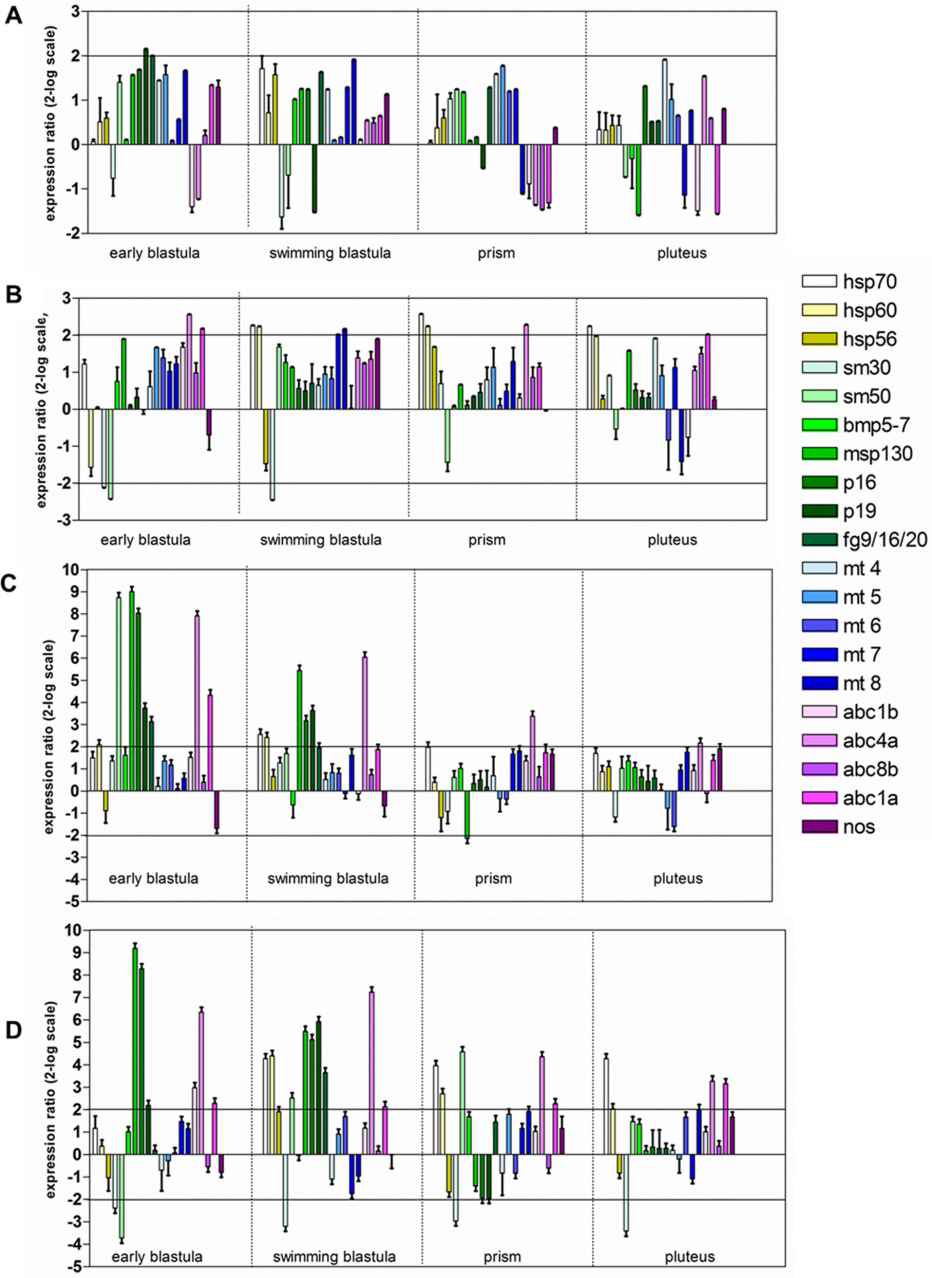
Gene expression analysis in the progeny of *P*. *lividus* females exposed to manganese 3.6 x 10^−5^ M for 2 days (A,B) and 9 days (C,D). The embryos were reared in sea water (A,C) and in manganese-containing sea water (B,D). Data are reported as a fold difference in the expression levels of the analyzed genes, compared to controls (mean ± SD), offspring of females kept during the whole experimental period in sea water without addition of metal. Fold differences equal or greater than ± 2 (see horizontal guidelines at values of 2 and—2) were considered significant. Experiments were repeated at least on 3 biological replicates.

Further experiments were performed to evaluate possible variation in the expression of genes involved in several processes, such as stress response (heat shock proteins *hsp70*, *hsp60* and *hsp56*), skeletogenesis (spicule matrix proteins *sm30*, *sm50* and *msp130*, the growth factor *bmp5-7*, the proteins involved in skeleton formation *p16* and *p19*, the fibroblast growth factor *fg9/16/20*), detoxification (metallothioneins *mt4*, *mt5*, *mt6*, *mt7* and *mt8*), multidrug efflux (abc transporter *abc1b*, *abc4a*, *abc8b*, *abc1a*) and NO production (*nos*). Their expression was followed at the different developmental stages in the offspring of females exposed to cadmium (Fig 4, S1 Table) and manganese (Fig 5, S1 Table) for 2 (A,B) and 9 days (C,D) and reared in sea water without metal (A,C) and in sea water containing metal (B,D). The data, obtained by Real Time qPCR, were normalized using as reference gene *Pl-Z12-1* and expressed with respect to the control values, offspring of females kept during the whole experimental period in sea water without addition of metals.

The offspring of females exposed to cadmium for both 2 and 9 days showed a great variation in gene expression when embryos were reared in sea water (Fig 4A and 4C, S1 Table) or in cadmium-containing sea water (Fig 4B and 4D, S1 Table), with respect to controls. In details, among the stress genes, *hsp70* was up-regulated in almost all developmental stages in the offspring of females exposed to cadmium for 2 or 9 days and reared both in sea water and in cadmium-containing sea water. The expression of *hsp60* and *hsp56* was up-regulated only at some developmental stages: *hsp60* at the early and swimming blastula (offspring of females exposed to cadmium for 2 days and reared in sea water and in cadmium-containing sea water, respectively) and *hsp56* at the swimming blastula and prism (offspring of females exposed to cadmium for 2 or 9 days and reared in metal-containing sea water, respectively). Among the skeletogenic genes, the spicule matrix protein 30, *sm30*, was up-regulated only at the early blastula and swimming blastula stages both in the offspring of females exposed for 2 days and reared in sea water and in cadmium-containing sea water. On the contrary, in the offspring of females exposed to cadmium for 9 days, this gene was down-regulated at all developmental stages when embryos were reared in cadmium-containing sea water and only at the prism and pluteus stages when development was performed in the absence of metal. Up-regulation of *sm50* was found only at the early blastula and swimming blastula stages in the offspring of females exposed to cadmium for 2 days and reared in sea water. A variation in the expression of *msp130* was mainly found in the offspring of females treated with cadmium for 2 or 9 days and reared in metal-containing water. The expression of *p16* and *p19* was also affected by the treatments. In the offspring of females exposed to cadmium for 2 days both genes were up-regulated at the initial stages (early and swimming blastula) and down-regulated at the prism and pluteus stages. The same trend (up-regulation at the initial stages and down-regulation at later stages) was observed for *p16* in the offspring of females exposed to cadmium for 9 days and reared in the presence of metal, whereas the expression of *p19* under these conditions increased only at the pluteus stage. The expression of *fg9/16/20* increased at early developmental stages in all experimental conditions. Among detoxification genes, the expression of metallothioneins increased to a different extent at almost all developmental stages in the offspring of females exposed to cadmium for 2 or 9 days and reared in metal-containing sea water. However, their expression was also up-regulated at some stages when development was performed in sea water without metal, except *mt4* which is down-regulated at the prism stage in the offspring of females exposed to cadmium for 2 days. Also the multidrug efflux genes showed variations in their expression following the different treatments. The transporters *abc1b* and *abc8b* were mostly down-regulated at different developmental stages and experimental conditions, except in some few conditions when they are up-regulated. The gene *abc1a* was always up-regulated at some developmental stages, whereas the expression of *abc4a* changed according to the time of adult exposure and developmental conditions. In the offspring of females exposed to cadmium for 2 days and reared in sea water or in metal-containing sea water the gene *abc4a* was up-regulated at the early and swimming blastula stages and down-regulated at late developmental stages. When females were exposed for 9 days, the offspring showed an up-regulation of this gene at all stages and conditions. The expression of *nos* was slightly affected only in the offspring of females exposed to cadmium for 2 days and reared in sea water and in cadmium-containing sea water.

In the presence of manganese, the greater effect on gene expression was found in the offspring after treatment with the metal for prolonged time (Fig 5, S1 Table). In detail, the expression of only *p19* was slightly affected in the offspring of females exposed for 2 days and reared in sea water (Fig 5A). When embryos were allowed to develop in manganese-containing seawater the expression of many genes was modified (Fig 5B). Among stress genes, *hsp70* was up-regulated at the swimming blastula, prism and pluteus stages, whereas *hsp60* only at the swimming blastula and prism stages. The expression of *sm30* decreased at the early and swimming blastula stages, while *sm50* was down-regulated only at the early blastula stage. Moreover, *mt7* and *mt8* were slightly up-regulated at the swimming blastula stage. Among the *abc* transporter, *abc4a* was up-regulated at the early blastula and prism stages and *abc1a* at the early blastula and pluteus stages. The offspring of females exposed for 9 days and reared in sea water showed an up regulation of some genes mostly at the early and swimming blastula stages. At the early blastula stage, *hsp60*, *sm50*, *msp130*, *p16*, *p19*, *fg9/16/20*, *abc4a* and *abc1a* were up-regulated. At the swimming blastula stage, *hsp70*, *hsp60*, *msp130*, *p16*, *p19* and *abc4a* increased their expression (Fig 5C). When embryos were reared in sea water containing manganese, the expression of these genes also changed (Fig 5D). However, under these conditions, *sm50* was down-regulated at the early blastula stage and up-regulated at the swimming blastula and prism stages. Moreover, *sm30* was down-regulated at all developmental stages and *mt8* and *abc1b* are slightly up-regulated at the pluteus and early blastula stages, respectively. The expression of *nos* was not modified by manganese treatment at all developmental stages analyzed.

## Discussion

The results of this study on the effects of cadmium and manganese exposure for 2 and 9 days on *P*. *lividus* females and their offspring greatly expanded previous investigations on the impact of toxic metals on adult sea urchins [36, 44–46], providing also information on the transmission of the “maternal” stress to the progeny at morphological, biochemical and molecular level.

Our findings that cadmium treatment impaired the ability of sea urchins to reproduce whereas manganese slightly affected the reproductive state indicated that the latter has “apparently” no toxic effects on adult sea urchins fertility. However, the exposure to both cadmium and manganese dramatically reduced the levels of NO in the ovaries. This signaling molecule has already been shown to mediate the response of sea urchin developing embryos to different stress agents [10, 34]. Therefore, it is likely that also manganese can affect female sea urchins defenses, although to a lesser extent than cadmium. These findings suggest that the measurements of the reproductive parameters, GSI, spawning and fertilization success, are not sufficient to assess the health of sea urchins, as also pointed out by Au et al. [45], whereas the determination of NO levels may represent an additional biomarker to monitor the wellness of the animals.

An important outcome of this study was provided by the analysis of the progeny of *P*. *lividus* females exposed to the metals and reared in sea water in the presence or in the absence of metals. Our findings clearly indicated that females exposed to cadmium and manganese produced an offspring with an high proportion of abnormal larvae also when the development was performed in normal sea water. As expected, the abnormalities increased with the time of female exposure and in the presence of the metal during embryo development. Phenotypic analysis of the progeny of females treated with environmentally relevant concentrations of cadmium and manganese confirmed previous results of a lower toxicity of manganese compared to cadmium [10,16]. Moreover, a general increase in NO levels especially at later developmental stages was found, similarly to the increase obtained by exposing directly developing embryos to the metals after fertilization [10]. However, an important result of this study was that with both cadmium and manganese, metal toxicity was exacerbated by maternal treatment. In fact, the percentage of abnormal plutei from treated females was much higher than those obtained when only embryos were exposed to metals [10]. This result suggested that maternal toxicity was transmitted to the progeny.

The present study provided first insights into the molecular mechanisms that mediate the toxic effects of cadmium and manganese on the progeny of exposed sea urchins. After maternal cadmium exposure for 2 days, significant changes in gene expression were found in developing embryos reared both in sea water with or without cadmium, whereas, after maternal manganese exposure for the same time, a significant modification in the expression of the selected genes was found only in developing embryos reared in sea water containing manganese. Our data, showing an up-regulation of the *hsps* in the offspring of females treated with cadmium and manganese, extend previous studies reporting the increase in hsps protein levels in larvae and adult cells or tissues of *P*. *lividus* after heat shock, X-ray, UVB, cadmium and manganese exposure [30, 47–51]. During the induction of heat shock proteins, cells are refractory to the toxicity of various agents and their protection is partially due to the inhibition of apoptosis [52]. The accumulation of damaged proteins acts as a signal for the induction of stress response and/or activation of the apoptotic program [53]. However, if the concentration of heat shock proteins is not enough to block the toxic effect of the metal, cells undergo to apoptosis [54]. Therefore, the up-regulation of *hsps* transcriptional levels confirms the activation of a defence mechanism to protect developing embryos against metals. Also the expression levels of some skeletogenic genes were modified by metal treatments. Sea urchin larvae have a simple skeleton consisting of a small number of spicules [55]. The skeleton is composed of magnesia calcite and spicule matrix proteins [56]. The cells responsible for the skeleton formation are the primary mesenchyme cells (PMCs), which synthesize specific marker proteins, including msp 130, a cell surface glycoprotein likely involved in calcium transport [57], sm30 encoding an acidic glycoprotein of the spicule matrix [58], sm50 encoding a basic non glycosylated protein [59], found in Golgi and small vesicles, p16 and p19, small proteins involved in the formation of the biomineralized skeleton in embryos and adults [60]. The observed variations in the expression of these genes may be due to the need to carry on the development under stressful conditions. In fact, the expression of the skeletogenic genes has been reported to be affected by other stressors, such as X-rays [50], high CO_2_ [61], heat shock [62], decadienal [34, 63], UVB exposure [49]. The finding that the expression level of the growth factor *bmp5-7* was not affected by cadmium or manganese treatment is in line with previous data showing that this gene is not involved during skeletogenesis [10,64]. Metals exposure also caused variations in the expression rate of the fibroblast growth factor *fg9/16/20*, coding for a protein involved in PMC migration and skeletal morphology, prefiguring the branching pattern of the skeleton. The inhibition of the production of these transcripts has a dramatic effect on sea urchin biomineralization because it leads to the absence of skeleton [65]. The observed up-regulation of this gene following cadmium and manganese treatment could prevent the inhibition of calcification in the PMCs, as also reported during CO_2_ exposure [66]. Metals are also responsible for the variation in the expression of genes which may be involved in detoxification processes such as metallothioneins and abc transporters. Our finding that the expression of *mts* increased in the offspring of females exposed to cadmium and manganese and reared in metal-containing sea water confirmed that elevated expression of these proteins was a hallmark of metal exposure [67]. The abc-transporters are a conserved family of membrane proteins that use ATP to move compounds across membranes in both adult and embryo. They can transport peptides, metals, xenobiotics and ions necessary for homeostasis, protection, and signalling [68]. Sea urchin embryos use different abc-transporters during the early developmental stages. The importance of the abc-transporters in the response to environmental stress was demonstrated by Kurelec [69]. In particular, the authors showed that abc-transporters can protect organisms from several pollutants. Several studies demonstrated the modulation of *abc* genes and transport activities. In piscine cell line, for example, after 24h of exposure to four different metals (CdCl_2_, HgCl_2_, As_2_O_3_ or K_2_Cr_2_O_7_) the *abcc2–4* genes were dose-dependently up-regulated by all metals, while *abcb1* and *abcc1* were less affected [70].

**Fig 6:**
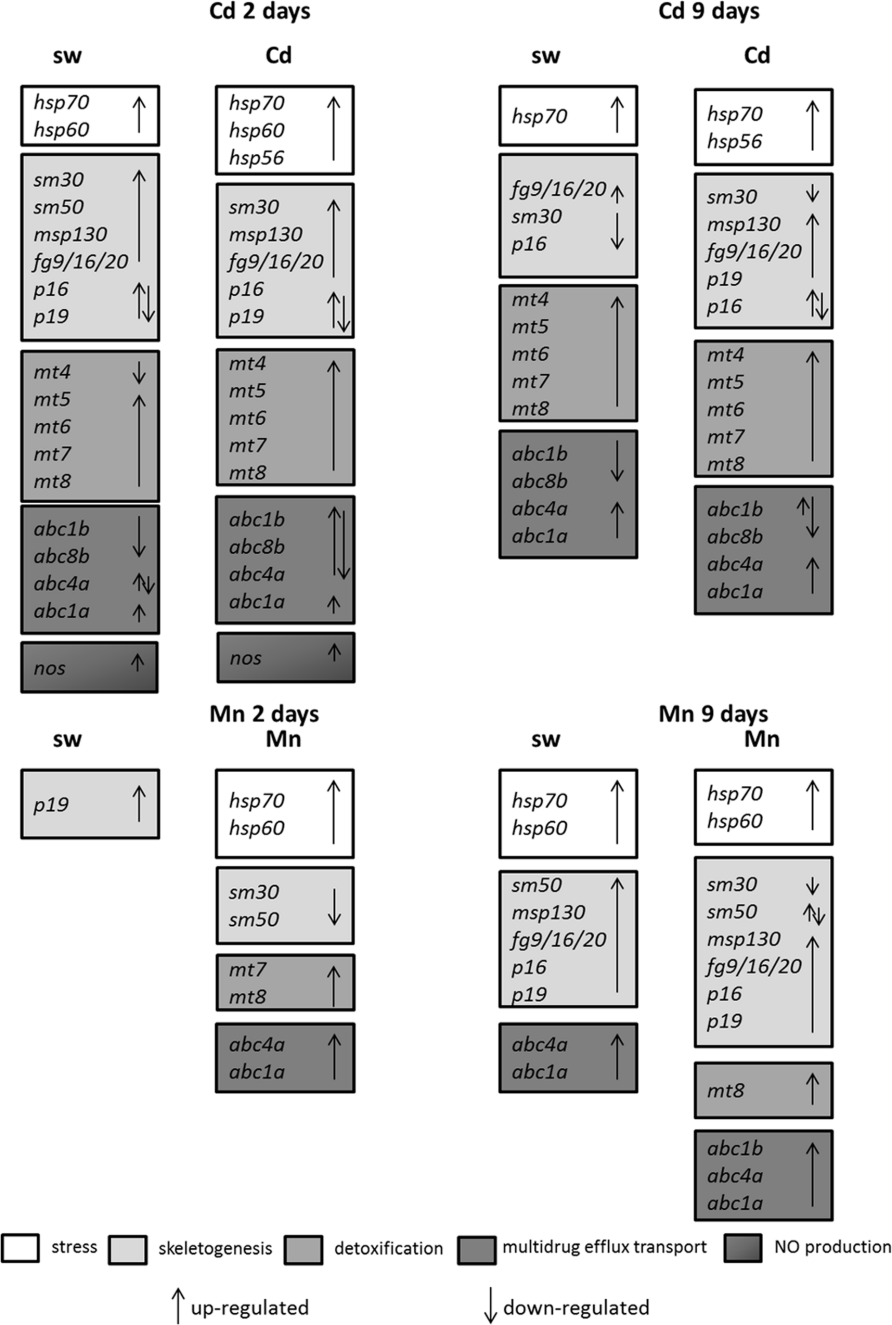
Synopsis of the patterns of up- and down-regulation of different classes of genes in the offspring of *P*. *lividus* females exposed to cadmium (Cd) or manganese (Mn) for 2 and 9 days. The two arrows indicate the up- or down-regulation of genes in different developmental stages.

Analysis of the different classes of genes revealed little variations in the number and type of genes affected in embryos reared in cadmium-containing sea water, compared to embryos reared in sea water without metal, at both times of exposure (Fig 6). The situation was different in the offspring of females treated with manganese. When females were exposed for short periods (2 days) almost all classes of genes were affected only when offspring was reared in sea water containing manganese. At longer time of treatments (9 days) also in the offspring reared in sea water without metals the majority of classes of genes was regulated. The different response at gene level of sea urchin developing embryos from females exposed to cadmium and manganese is correlated to the different nature of these metals. Indeed, cadmium is a known heavy metal, toxic even at very low concentrations [71] and without any biological role, whereas manganese is a naturally occurring metal required in trace amounts by the organisms, toxic only at high levels. These results again confirmed the higher toxicity of cadmium compared to manganese. On the other hand, after maternal treatments for longer periods (9 days), the offspring of females exposed to both cadmium and manganese showed a great variation in the expression of the majority of the selected genes, indicating that the time of maternal exposure affects the molecular response of the offspring. However, considering that gene expression is only a part of the cascade processes leading to the elevated expression of a protein, future studies should be directed to obtain a clear picture of the dynamics of the defensome systems in *P*. *lividus* by investigating the protein expression patterns.

The variations in gene expression in the offspring of treated females could be correlated to changes in the endogenous levels of NO that, as we have previously shown [10], are able to affect gene expression in sea urchin developing embryos treated with cadmium and manganese after fertilization. In fact, a significant increase in NO production was found in the different developmental stages of the offspring derived from exposed females. The increase of NO could derive both by stimulation of nos activity e.g. by intracellular calcium concentration [72] or by regulation at the transcriptional level. Indeed, the slight up-regulation of *nos* at early developmental stages in the progeny of females exposed to cadmium for 2 days and the increase of NO levels at late stages may be explained with an early transcriptional regulation of *nos* gene, responsible for the later NO production. However, further investigation should be performed, by using NOS inhibitors and NO donors, to evaluate the differences found in embryos treated after fertilization [10] and the offspring of treated females (this work). Interestingly, *nos* was up-regulated in the offspring only when the metal treatment was performed on the females and not when fertilized eggs were treated with metals [10]. One speculation could be related to the presence of a sensitization mechanism in the offspring of treated females, in which *nos* is fast up-regulated in response to the metal.

In conclusion, our finding that cadmium and manganese, at environmentally relevant concentrations (10^−6^ M and 3.6 x 10^−5^ M, respectively) negatively affected both *P*. *lividus* females reproductivity and development of their progeny is of considerable ecological relevance, considering that maternal exposure to contaminants can impair reproduction and on a large-scale and long-term can affect recruitment of sea urchin populations habiting areas potentially exposed to contaminants (e.g. coastal or industrial zone).

## Supporting Information

S1 Table Variation in gene expression in the offspring of females exposed to cadmium and manganese. Different developmental stages of the offspring of females exposed to cadmium (Cd) and manganese (Mn) for 2 and 9 days and reared in sea water (SW) and in metal-containing SW were examined for gene expression. Values (mean ± SD) equal or greater than ± 2 are reported as a fold difference in the expression levels of the analyzed genes, compared to controls, offspring of females kept during the whole experimental period in sea water without addition of metal. Experiments were repeated at least on 3 biological replicates.
(DOC)

## References

[1] Fan AM. An introduction to monitoring and environmental and human risk assessment of metal In: Magos L, Suzuki T, editors. Toxicology of Metals. CRC Lewis Publishers: Boca Raton; 1996 pp. 5–9.

[2] Perez-Lopez M, Alonso J, Novoa-Valinas MC, Melgar MJ. Assessment of heavy metal contamination of seawater and marine limpet, *Patella vulgata* L., from Northwest Spain. J Environ Sci Health A Tox Hazard Subst Environ Eng. 2003; 38: 2845–2856. 14672319 10.1081/ese-120025835

[3] Roesijadi G, Robinson WE. Metal regulation in aquatic animals: mechanisms of uptake, accumulation, and release In: Malins DC, Ostrander GK, editors. Aquatic Toxicology-Molecular, Biochemical, and Cellular Perspectives. Lewis Publishers: Boca Raton; 1994 pp. 387–420.

[4] Migliaccio O, Castellano I, Romano G, Palumbo A. Response of sea urchin to environmental stress In: Banks ER, editor. Sea Urchins: Habitat, Embryonic development and importance in the environment. Nova Science Publishers, Inc; 2014a pp. 1–23.

[5] Roccheri MC, Matranga V. Cellular, biochemical and molecular effects of cadmium on *Paracentrotus lividus* sea urchin development In: Parvau R G, editor. Cadmium in the environment. Nova Science Publishers, Inc; 2009 pp. 1–30.

[6] Pinsino A, Matranga V, Trinchella F, Roccheri MC. Sea urchin embryos as an in vivo model for the assessment of manganese toxicity: developmental and stress response effects. Ecotoxicology. 2010; 19: 555–562. 10.1007/s10646-009-0432-0 19882348

[7] Pinsino A, Roccheri MC, Costa C, Matranga V. Manganese interferes with calcium, perturbs ERK signaling and produces embryos with no skeleton. Toxicol Sci. 2011; 23: 217–230. 10.1093/toxsci/kfr152 21659617

[8] Vaschenko MA, Luchsheva LN, Zhadan PM, Belcheva NN, Syasina IG Silina AV. Assessment of the ecological situation in Alekseev Bight (Peter the Great Bay, Sea of Japan) by biological and biochemical parameters. Biol Morya. 1999; 25: 96–97.

[9] Filosto S, Roccheri MC, Bonaventura R, Matranga V. Environmentally relevant cadmium concentrations affect development and induce apoptosis of *Paracentrotus lividus* larvae cultured in vitro. Cell Biol Toxicol. 2008; 24: 603–610. 10.1007/s10565-008-9066-x 18322810

[10] Migliaccio O, Castellano I, Romano G, Palumbo A. Stress response to cadmium and manganese in *Paracentrotus lividus* developing embryos is mediated by nitric oxide. Aquat Toxicol. 2014b; 156: 125–134. 25181703 10.1016/j.aquatox.2014.08.007

[11] Kremling K, Streu P. Behaviour of dissolved Cd, Co, Zn, and Pb in North Atlantic near-surface waters (30° N/60° W to 60° N/2° W). Deep Sea Res I. 2001; 12: 2541–2567.

[12] Pohl C, Hennings U. The coupling of long-term trace metal trends to seasonal diffusive trace metal fluxes at the oxic–anoxic interface in the Gotland Basin (57°19.20′ N; 20° 03.00′ E) Baltic Sea. J Mar Sys. 2005; 56: 207–225.

[13] Kobayashi N, Okamura H. Effects of heavy metals on sea urchin embryo development. 1. Tracing the cause by the effects. Chemosphere. 2004; 55: 1403–1412. 15081783 10.1016/j.chemosphere.2003.11.052

[14] Tankere SPC, Statham PJ. Distribution of dissolved Cd, Cu, Ni and Zn in the Adriatic Sea. Mar Pollut Bull. 1996; 32: 623–630.

[15] Beiras R, Fernandez N, Bellas J, Besada V, Gonzalez-Quijano A, Nunes T. Integrative assessment of marine pollution in Galician estuaries using sediment chemistry, mussel bioaccumulation, and embryo-larval toxicity bioassays. Chemosphere. 2003; 52: 1209–1224. 12821002 10.1016/S0045-6535(03)00364-3

[16] Kobayashi N, Okamura H. Effects of heavy metals on sea urchin embryo development. Part 2. Interactive toxic effects of heavy metals in synthetic mine effluents. Chemosphere. 2005; 61: 1198–1203. 16263390 10.1016/j.chemosphere.2005.02.071

[17] Fatoki OS, Mathabatha S. An assessment of heavy metal pollution in the East London and Port Elizabeth harbours. Water SA. 2001; 27: 233–240.

[18] ATSDR. Draft Toxicological Profile for Manganese Agency for Toxic Substances and Disease Registry. Division of Toxicology and Environmental Medicine/ Applied Toxicology Branch, Atlanta, Georgia 2008.

[19] Santamaria AB. Manganese exposure, essentiality and toxicity. Indian J Med Res. 2008; 128: 484–500. 19106442

[20] Daly MJ. A new perspective on radiation resistance based on *Deinococcus radiodurans* . Nat Rev Microbiol. 2009; 7: 237–244. 10.1038/nrmicro2073 19172147

[21] CICAD. Manganese and its Compounds: Environmental Aspects In: Concise International Chemical Assessment Document, vol. 63 WHO, Geneva, Switzerland; 2004.

[22] Trefry JH, Presley BJ, Keeney-Kennicutt WL, Trocine RP. Distribution and chemistry of manganese, iron, and suspended particulates in orca basin. Geo-Mar Lett. 1984; 4: 125–130.

[23] Aller RC. The sedimentary Mn cycle in long island sound: its role as intermediate oxidant and the influence of bioturbation, O_2_, and C_org_ flux on diagenetic reaction balances. J Mar Res. 1994; 52: 259–295.

[24] Pagano G, Cipollaro M, Corsale G, Esposito A, Ragucci E. The Sea Urchin: Bioassay for the Assessment of damage from environmental contaminants. In: Philadelphia American Society for testing and materials. 1986. pp. 67–92.

[25] Warnau M, Biondo R, Temara A, Bouquegneau JM, Jangoux M, Dubois P. Distribution of heavy metal in the echinoid *Paracentrotus lividus* (Lmk) from the Mediterranean *Posidonia oceanica* ecosystem: seasonal and geographical variations. J Sea Res. 1998; 39: 267–280.

[26] Harmelin JG, Bouchon C, Hong JS. Impact de la pollution sur la distribution des échinodermes des substrats durs en Provence (Méditerranée Nord-occidentale). Téthys. 1981; 10: 13–36.

[27] Bayed A, Quiniou F, Benrha A, Guillou M. The *Paracentrotus lividus* populations from the northern Moroccan Atlantic coast: growth, reproduction and health condition. J Mar Biol Assoc U K. 2005; 85: 999–1007.

[28] Soualili D, Dubois P, Gosselin P, Pernet P, Guillou M. Assessment of seawater pollution by heavy metals in the neighbourhood of Algiers: use of the sea urchin, *Paracentrotus lividus*, as a bioindicator. ICES J Mar Sci. 2008; 65: 132–139.

[29] Russo R, Bonaventura R, Zito F, Schroeder HC, Müller I, Müller WEG, et al Stress to cadmium monitored by metallothionein gene induction in *Paracentrotus lividus* embryos. Cell Stress Chaperones. 2003; 8: 232–241. 14984056 10.1379/1466-1268(2003)008<0232:stcmbm>2.0.co;2 PMC514876

[30] Roccheri MC, Agnello M, Bonaventura R, Matranga V. Cadmium induces the expression of specific stress proteins in sea urchin embryos. Biochem Biophys Res Commun. 2004; 321: 80–87. 15358218 10.1016/j.bbrc.2004.06.108

[31] Agnello M, Filosto S, Scudiero R, Rinaldi AM, Roccheri MC. Cadmium induces an apoptotic response in sea urchin embryos. Cell Stress Chaperones. 2007; 12: 44–50. 17441506 10.1379/CSC-229R.1 PMC1852892

[32] Pinsino A, Roccheri MC, Matranga V. Manganese overload affects p38 MAPK phosphorylation and metalloproteinase activity during sea urchin embryonic development. Mar Environ Res. 2013; 93: 64–69. 10.1016/j.marenvres.2013.08.004 23998794

[33] Chiarelli R, Agnello M, Bosco L, Roccheri MC. Sea urchin embryos exposed to cadmium as an experimental model for studying the relationship between autophagy and apoptosis. Mar Environ Res. 2014; 93: 47–55. 10.1016/j.marenvres.2013.06.001 23838188

[34] Romano G, Costantini M, Buttino I, Ianora A, Palumbo A. Nitric oxide mediates the stress response induced by diatom aldehydes in the sea urchin *Paracentrotus lividus* . PLoS One. 2011; 6: e25980 10.1371/journal.pone.0025980 22022485 PMC3191173

[35] Vaschenko MA, Zhadan PM, Medvedeva LA. Developmental disturbances in sea urchin *Strongylocentrotus intermedius* larvae from polluted regions of Peter the Great Bay, Sea of Japan. Biol Morya. 1999; 20: 137–147.

[36] Vaschenko MA, Zhadan PM. Developmental disturbances in the progeny of sea urchins as an index of environmental pollution. Russ J Ecol. 2003; 34: 418–424.

[37] Naidenko T. Abnormality of development in *Strongylocentrotus intermedius* (A. Agassiz) larvae from polluted habitat in Amursky Bay, Peter the Great Bay. Pub Seto Mar Biol Lab. 1997; 38: 1–11.

[38] Quiniou F, Guillou M, Judas A. Arrest and delay in embryonic development in sea urchin populations of the bay of Brest (Brittany, France): link with environmental factors. Mar Poll Bull. 1999; 38: 401–406.

[39] Radenac G, Fichet D, Miramand P. Bioaccumulation and toxicity of four dissolved metals in *Paracentrotus lividus* sea-urchin embryo. Mar Environ Res. 2001; 51: 151–166. 11468814 10.1016/s0141-1136(00)00092-1

[40] Carballeira C, Ramos-Gómez J, Martín-Díaz L, DelValls TA. Identification of specific malformations of sea urchin larvae for toxicity assessment: Application to marine pisciculture effluents. Mar Environ Res. 2012; 77: 12–22. 10.1016/j.marenvres.2012.01.001 22341183

[41] Green LC, Wagner DA, Glogowski J. Analysis of nitrate, nitrite and nitrate in biological fluids. Anal Biochem. 1982; 126: 131–138. 7181105 10.1016/0003-2697(82)90118-x

[42] Ragusa MA, Costa S, Gianguzza M, Roccheri MC, Gianguzza F. Effect of cadmium exposure on sea urchin development assessed by SSH and RT-qPCR: metallothionein genes in marine invertebrates: focus on *Paracentrotus lividus* sea urchin development. Mol Biol Rep. 2013; 40: 2157–2167. 10.1007/s11033-012-2275-7 23212613

[43] Pfaffl MW, Horgan GW, Dempfle L. Relative expression software tool (REST) for group-wise comparison and statistical analysis of relative expression results in real-time PCR. Nucleic Acids Res. 2002; 30: e36 11972351 10.1093/nar/30.9.e36 PMC113859

[44] Au DW, Lee CY, Chan KL, Wu RS. Reproductive impairment of sea urchins upon chronic exposure to cadmium. Part I: Effects on gamete quality. Environ Pollut. 2001a; 111: 1–9. 11202702 10.1016/s0269-7491(00)00035-x

[45] Au DW, Lee CY, Chan KL, Wu RS. Reproductive impairment of sea urchins upon chronic exposure to cadmium. Part II: Effects on sperm development. Environ Pollut. 2001b; 111: 11–20. 11202702 10.1016/s0269-7491(00)00035-x

[46] Manzo S, Buono S, Cremisini C. Cadmium, lead and their mixtures with copper: *Paracentrotus lividus* embryotoxicity assessment, prediction, and offspring quality evaluation. Ecotoxicology. 2010; 19: 1209–1223. 10.1007/s10646-010-0506-z 20552397

[47] Giudice G, Sconzo G, Roccheri MC. Studies on heat shock protein in sea urchin development. Dev Growth Differ. 1999; 41: 375–380. 10466924 10.1046/j.1440-169x.1999.00450.x

[48] Roccheri MC, Patti M, Agnello M, Gianguzza F, Carra E, Rinaldi AM. Localization of Mitochondrial Hsp58 chaperonine during sea urchin development. Biochem Biophys Res Commun. 2001; 287: 1093–1098. 11587534 10.1006/bbrc.2001.5503

[49] Bonaventura R, Poma V, Costa C, Matranga V. UVB radiation prevents skeleton growth and stimulates the expression of stress markers in sea urchin embryos. Biochem Biophys Res Commun. 2005; 328: 150–157. 15670763 10.1016/j.bbrc.2004.12.161

[50] Matranga V, Zito F, Costa C, Bonaventura R, Giarrusso S, Celi F. Embryonic development and skeletogenic gene expression affected by X-rays in the Mediterranean sea urchin *Paracentrotus lividus* . Ecotoxicology. 2010; 19: 530–537. 10.1007/s10646-009-0444-9 19943107

[51] Bonaventura R, Zito F, Costa C, Giarrusso S, Celi F, Matranga V. Stress response gene activation protects sea urchin embryos exposed to X-rays. Cell Stress Chaperones. 2011; 16: 681–687. 10.1007/s12192-011-0277-3 21720812 PMC3220391

[52] Samali A, Cotter TG. Heat shock proteins increase resistance to apoptosis. Exp Cell Res. 1996; 223: 163–170. 8635489 10.1006/excr.1996.0070

[53] Latchman DS. Stress proteins: An overview. Handb Exp Pharmacol. 1999; 136: 1–7.

[54] Hamada T, Tanimoto A, Sasaguri Y. Apoptosis induced by cadmium. Apoptosis. 1997; 2: 359–367. 14646532 10.1023/a:1026401506914

[55] Ameye L, Hermann R, Killian C, Wilt F, Dubois P. Ultrastructural localization of proteins involved in sea urchin biomineralization. J Histochem Cytochem. 1999; 47: 1189–1200. 10449540 10.1177/002215549904700911

[56] Wilt FH. Developmental biology meets materials science: morphogenesis of biomineralized structures. Dev Biol. 2005; 280: 15–25. 15766744 10.1016/j.ydbio.2005.01.019

[57] Anstrom JA, Chin JE, Leaf DS, Parks AL, Raff RA. Localization and expression of msp 130 a primary mesenchyme lineage specific cell surface protein of the sea urchin embryo. Development. 1987; 101: 255–265. 3128442 10.1242/dev.101.2.255

[58] George C, Killian CE, Wilt FH. Characterization and expression of a gene encoding a 30.6 kDa *Strongylocentrotus purpuratus* spicule matrix protein. Dev Biol. 1991; 147: 334–342. 1717322 10.1016/0012-1606(91)90291-a

[59] Killian CE, Wilt FH. Characterization of the proteins comprising the integral matrix of *Strongylocentrotus purpuratus* embryonic spicules. J Biol Chem. 1997; 271: 9150–9159. 10.1074/jbc.271.15.9150 8621567

[60] Costa C, Karakostis K, Zito F, Matranga V. Phylogenetic analysis and expression patterns of p16 and p19 in *Paracentrotus lividus* embryos. Dev Genes Evol. 2012; 222: 245–251. 10.1007/s00427-012-0405-9 22565340

[61] Todgham AE, Hofmann GE. Transcriptomic response of sea urchin larvae *Strongylocentrotus purpuratus* to CO_2_-driven seawater acidification. J Exp Biol. 2009; 212: 2579–2594. 10.1242/jeb.032540 19648403

[62] Runcie D, Garfield DA, Babbitt CC, Wygoda JA, Mukjerjee S, Wray GA. Genetics of gene expression responses to temperature stress in a sea urchin gene network. Mol Ecol. 2012; 21: 4547–4562. 10.1111/j.1365-294X.2012.05717.x 22856327 PMC3866972

[63] Marrone V, Piscopo M, Romano G, Ianora A, Palumbo A, Costantini M. Defensome against toxic diatom aldehydes in the sea urchin *Paracentrotus lividus* . PLoS One. 2012; 7: e31750 10.1371/journal.pone.0031750 22363721 PMC3282763

[64] Zito F, Costa C, Sciarrino S, Poma V, Russo R. Expression of univin, a TGF-b growth factor, requires ectoderm-ECM interaction and promotes skeletal growth in the sea urchin embryo. Dev Biol. 2003; 264: 217–227. 14623243 10.1016/j.ydbio.2003.07.015

[65] Röttinger E, Saudemont A, Duboc V, Besnardeau L, McClay D, Lepage T. FGF signals guide migration of mesenchymal cells, control skeletal morphogenesis and regulate gastrulation during sea urchin development. Development. 2008; 135: 353–365. 18077587 10.1242/dev.014282

[66] Martin S, Richier S, Pedrotti ML, Dupont S, Castejon C, Gerakis Y, et al Early development and molecular plasticity in the Mediterranean sea urchin *Paracentrotus lividus* exposed to CO_2_-driven acidification. J Exp Biol. 2011; 214: 1357–1368. 10.1242/jeb.051169 21430213

[67] Amiard JC, Amiard-Triquet C, Barka S, Pellerin J, Rainbow PS. Metallothioneins in aquatic invertebrates: their role in metal detoxification and their use as biomarkers. Aquat Toxicol. 2006; 76: 160–202. 16289342 10.1016/j.aquatox.2005.08.015

[68] Leslie EM, Deeley RG, Cole SP. Toxicological relevance of the multidrug resistance protein 1, MRP1 (ABCC1) and related transporters. Toxicology. 2001; 167: 13–23. 10.1016/s0300-483x(01)00454-1 11557126

[69] Kurelec B. The Multixenobiotic Resistance Mechanism in Aquatic Organisms. Crit Rev Toxicol. 1992; 22: 23–43. 1352103 10.3109/10408449209145320

[70] Della Torre C, Zaja R, Loncar J, Smital T, Focardi S, Corsi I. Interaction of ABC transport proteins with toxic metals at the level of gene and transport activity in the PLHC-1 fish cell line. Chem Biol Interact. 2012; 198: 1–17. 10.1016/j.cbi.2012.04.008 22580103

[71] Foulkes EC. Transport of toxic metal across cell membranes. Proc Soc Exp Biol Med. 2000; 223: 234–240. 10719835 10.1046/j.1525-1373.2000.22334.x

[72] Griffith OW, Stuehr DJ. Nitric oxide synthases: properties and catalytic mechanism. Annu Rev Physiol. 1995; 57: 707–736. 7539994 10.1146/annurev.ph.57.030195.003423

