# Supplementary material for: Maternal Exposure to Cadmium and Manganese Impairs Reproduction and Progeny Fitness in the Sea Urchin Paracentrotus lividus
Source: PLoS One. 2015 Jun 30;10(6):e0131815. doi: 10.1371/journal.pone.0131815 (PMC4488381; doi:10.1371/journal.pone.0131815)
Supplement: S1 Table — Different developmental stages of the offspring of females exposed to cadmium (Cd) and manganese (Mn) for 2 and 9 days and reared in sea water (SW) and in metal-containing SW were examined for gene expression. Values (mean ± SD) equal or greater than ± 2 are reported as a fold difference in the expression levels of the analyzed genes, compared to controls, offspring of females kept during the whole experimental period in sea water without addition of metal. Experiments were repeated at least on 3 biological replicates. (DOC) [file pone.0131815.s001.doc]

|  | **Early blastula** | **Swimming blastula** | **Prism** | **Pluteus** |
| --- | --- | --- | --- | --- |
| ***hsp70*** |  |  |  |  |
| Cd 2 d SW | +3.09 ± 0.293 | -- | +3.61± 0.291 | +2.51± 0.287 |
| Cd 2 d Cd | +3.66± 0.287 | +3.41± 0.292 | +4.24± 0.291 | +3.94± 0.298 |
| Cd 9 d SW | +3.18± 0.169 | +2.18± 0.171 | -- | +2.71± 0.165 |
| Cd 9d Cd | +3.6± 0.23 | +3.73± 0.159 | +4.18± 0.18 | +4.6± 0.21 |
| ***hsp60*** |  |  |  |  |
| Cd 2d SW | +5.27± 0.312 | +3.8± 0.276 | -- | -- |
| Cd 2d Cd | +3.58± 0.24 | +3.86± 0.176 | -- | -- |
| Cd 9d SW | -- | -- | -- | -- |
| Cd 9d Cd | -- | -- | -- | -- |
| ***hsp56*** |  |  |  |  |
| Cd 2d SW | -- | -- | -- | -- |
| Cd 2d Cd | -- | +3.1± 0.3 | -- | -- |
| Cd 9d SW | -- | -- | -- | -- |
| Cd 9d Cd | -- | -- | +2.63± 0.169 |  |
| ***sm30*** |  |  |  |  |
| Cd 2d SW | +2.37± 0.258 | +2.72± 0.313 | -- | -- |
| Cd 2d Cd | +2.17± 0.186 | +3.64± 0.312 | -- | -- |
| Cd 9d SW | -- | -- | -2.46± 0.195 | -2.88± 0.245 |
| Cd 9d Cd | -2.58± 0.193 | -2.7± 0.187 | -2.6± 0.107 | -3.9± 0.169 |
| ***sm50*** |  |  |  |  |
| Cd 2d SW | +2.54± 0.296 | +3.24± 0.3 | -- | -- |
| Cd 2d Cd | -- | -- | -- | -- |
| Cd 9d SW | -- | -- | -- | -- |
| Cd 9d Cd | -- | -- | -- | -- |
| ***bmp5-7*** |  |  |  |  |
| All conditions | -- | -- | -- | -- |
| ***msp130*** |  |  |  |  |
| Cd 2d SW | -- | -- | +2.5± 0.502 | -- |
| Cd 2d Cd | -- | +2.85± 0.334 | +2.79± 0.156 | +3.35± 0.27 |
| Cd 9d SW | -- | -- | -- | -- |
| Cd 9d Cd | +3.29± 0.108 | +2.71± 0.198 | +2.2± 0.154 | -- |
| ***p16*** |  |  |  |  |
| Cd 2d SW | -- | +4.6± 1.25 | -3.4± 0.518 | -- |
| Cd 2d Cd | +5.94± 0.876 | +2.78± 0.298 | -2.85± 0.168 | -- |
| Cd 9d SW | -- | -- | -2.11± 0.147 | -- |
| Cd 9d Cd | +3.2± 0.137 | +3.71± 0.198 | -3.7± 0.543 | -3± 0.154 |
| ***p19*** |  |  |  |  |
| Cd 2d SW | -- | +2.39± 0.629 | -6.1± 0.403 | -8.91± 0.254 |
| Cd 2d Cd | -- | +5.61± 0.476 | -6.296 | -6± 0.365 |
| Cd 9d SW | -- | -- | -- | -- |
| Cd 9d Cd | -- | -- | -- | +2.6± 0.428 |
| ***fg9/16/20*** |  |  |  |  |
| Cd 2dSW | +4.1± 0.154 | -- | -- | -- |
| Cd 2d Cd | +2.27± 0.259 | -- | -- | -- |
| Cd 9d SW | +2.2± 0.293 | +2± 0.443 | -- | -- |
| Cd 9d Cd | +3.25± 0.169 | -- | -- | -- |
| ***mt4*** |  |  |  |  |
| Cd 2d SW | -- | -- | -2.61± 0.682 | -- |
| Cd 2d Cd | +2.6± 0.499 | +2.48± 0.189 | +2.93± 0.512 | +4.02± 0.29 |
| Cd 9d SW | +2.98± 0.169 | +3.06± 0.543 | -- | +2.5± 0.009 |
| Cd 9d Cd | +3.71± 0.521` | +4.29± 0.122 | +5.6± 0.332 | +5.92± 0.787 |
| ***mt5*** |  |  |  |  |
| Cd 2d SW | -- | -- | +2.1± 0.111 | -- |
| Cd 2d Cd | +3.46± 0.341 | +4.23± 0.511 | +2.46± 0.29 | +2.99± 0.635 |
| Cd 9d SW | -- | -- | +2.7± 0.167 | +2.06± 0.166 |
| Cd 9d Cd | +2.61± 0.332 | +5.59± 0.112 | +3.18± 0.453 | +2.93± 0.302 |
| ***mt6*** |  |  |  |  |
| Cd 2d SW | -- | -- | +2.47± 0.178 | -- |
| Cd 2d Cd | -- | +2.90± 0.187 | -- | +2.64± 0.212 |
| Cd 9d SW | -- | +2.06± 0.111 | +3.29± 0.186 | +3.27± 0.312 |
| Cd 9d Cd | -- | +2.21± 0.09 | +4.7± 0.121 | +2.72± 0.312 |
| ***mt7*** |  |  |  |  |
| Cd 2d SW | +2.21± 0.296 | -- | -- | -- |
| Cd 2d Cd | +2.58± 0.234 | -- | +2.3± 0.545 | +2.1± 0.078 |
| Cd 9d SW | -- | -- | +2.21± 0.137 | -- |
| Cd 9d Cd | -- | -- | +2.83± 0.876 | +2.16± 0.178 |
| ***mt8*** |  |  |  |  |
| Cd 2d SW | +2.05± 0.298 | +2.08± 0.189 | +2.32± 0.177 | -- |
| Cd 2d Cd | +2.32± 0.119 | +2.98± 0.436 | -- | +2.34± 0.651 |
| Cd 9d SW | -- | -- | +2.64± 0.593 | +3.31± 0.235 |
| Cd 9d Cd | -- | -- | +2.95± 0.193 | +4.31± 0.893 |
| ***abc1b*** |  |  |  |  |
| Cd 2d SW | -2.38± 0.293 | -- | -- | -5.17± 0.485 |
| Cd 2d Cd | -3.7± 0.424 | +2.39± 0.158 | -2.52± 0.335 | -2.9± 0.296 |
| Cd 9d SW | -2.06± 0.097 | -2.08± 0.178 | -- | -- |
| Cd 9d Cd | -3.18± 0.168 | -3.07± 0.255 | -4.06± 0.675 | +2.02± 0.222 |
| ***abc4a*** |  |  |  |  |
| Cd 2d SW | +3.53± 0.255 | +4.66± 0.387 | -2.93± 0.187 | -5.23± 0.454 |
| Cd 2d Cd | +4.19± 0.169 | +6.49± 0.296 | -- | -2.2± 0.389 |
| Cd 9d SW | +3.19± 0.179 | +2.6± 0.169 | +2.72± 0.169 | -- |
| Cd 9d Cd | +2.3± 0.169 | +3.6± 0.206 | +3.04± 0.169 | +3.06± 0.189 |
| ***abc8b*** |  |  |  |  |
| Cd 2d SW | -- | -- | -2.75± 0.297 | -4.67± 0.245 |
| Cd 2d Cd | -3.5± 0.278 | +2.01± 0.02 | -2.91± 0.452 | -- |
| Cd 9d SW | -- | -- | -2.48± 0.32 | -3.18± 0.147 |
| Cd 9d Cd | -3.48± 0.623 | -- | -- | -5.58± 0.145 |
| ***abc1a*** |  |  |  |  |
| Cd 2d SW | -- | +2.95± 0.958 | -- | -- |
| Cd 2d Cd | -- | +5.09± 0.398 | +2.95± 0.199 | +2.78± 0.364 |
| Cd 9d SW | -- | +2.71± 0.159 | -- | -- |
| Cd 9d Cd | +2.49± 0.119 | +5.18± 0.455 | +3.2± 0.232 | -- |
| ***Nos*** |  |  |  |  |
| Cd 2d SW | -- | +2.05± 0.634 | -- | -- |
| Cd 2d Cd | +2.57± 0.296 | -- | -- | -- |
| Cd 9d SW | -- | -- | -- | -- |
| Cd 9d Cd | -- | -- | -- | -- |

|  | **Early blastula** | **Swimming blastula** | **Prism** | **Pluteus** |
| --- | --- | --- | --- | --- |
| ***hsp70*** |  |  |  |  |
| Mn 2d SW | -- | -- | -- | -- |
| Mn 2d Mn | -- | +2.25 ± 0.025 | +2.56 ± 0.155 | +2.24 ± 0.055 |
| Mn 9d SW | -- | +2.57 ± 0.38 | -- | -- |
| Mn 9d Mn | -- | +4.27 ± 0.381 | +3.96± 0.143 | +4.26 ± 0.358 |
| ***hsp60*** |  |  |  |  |
| Mn 2d SW | -- | -- | -- | -- |
| Mn 2d Mn | -- | +2.23± 0.025 | +2.23± 0.05 | -- |
| Mn 9d SW | +2.08± 0.355 | +2.42± 0.255 | -- | -- |
| Mn 9d Mn | -- | +4.4 ± 0.265 | +2.71± 0.432 | +2.03± 0.325 |
| ***hsp56*** |  |  |  |  |
| All conditions | -- | -- | -- | -- |
| ***sm30*** |  |  |  |  |
| Mn 2d SW | -- | -- | -- | -- |
| Mn 2d Mn | -2.10± 0.05 | -2.44± 0.132 | -- | -- |
| Mn 9d SW | -- | -- | -- | -- |
| Mn 9d Mn | -2.39± 0.368 | -3.2± 0.245 | -2.96± 0.380 | -3.41± 0.452 |
| ***sm50*** |  |  |  |  |
| Mn 2d SW | -- | -- | -- | -- |
| Mn 2d Mn | -2.41 ± 0.123 | -- | -- | -- |
| Mn 9d SW | +8.74± 0.386 | -- | -- | -- |
| Mn 9d Mn | -3.72± 0.756 | +2.53± 0.365 | +4.58± 0.521 |  |
| ***bmp5-7*** |  |  |  |  |
| All conditions | -- | -- | -- | -- |
| ***msp130*** |  |  |  |  |
| Mn 2d SW | -- | -- | -- | -- |
| Mn 2d Mn | -- | -- | -- | -- |
| Mn 9d SW | +9.01± 0.315 | +5.45± 0.365 | -2.14± 0.419 | -- |
| Mn 9d Mn | +9.19± 0.389 | +5.49± 0.298 |  | -- |
| ***p16*** |  |  |  |  |
| Mn 2d SW | -- | -- | -- | -- |
| Mn 2d Mn | -- | -- | -- | -- |
| Mn 9d SW | +8.03± 0.38 | +3.17± 0.368 | -- | -- |
| Mn 9d Mn | +8.28± 0.245 | +5.12± 0.198 | -- | -- |
| ***p19*** |  |  |  |  |
| Mn 2d SW | +2.15± 0.024 | -- | -- | -- |
| Mn 2d Mn | -- | -- | -- | -- |
| Mn 9d SW | +3.74± 0.332 | +3.64± 0.356 | -- | -- |
| Mn 9d Mn | +2.19± 0.299 | +5.92± 0.412 | -- | -- |
| ***fg9/16/20*** |  |  |  |  |
| Mn 2dSW | -- | -- | -- | -- |
| Mn 2d Mn | -- | -- | -- | -- |
| Mn 9d SW | +3.13± 0.378 | -- | -- | -- |
| Mn 9d Mn | -- | +3.64± 0.398 | -- | -- |
| ***mt4,mt5,mt6*** |  |  |  |  |
| All conditions | -- | -- | -- | -- |
| ***mt7*** |  |  |  |  |
| Mn 2d SW | -- | -- | -- | -- |
| Mn 2d Mn | -- | +2.014 ± 0.027 | -- | -- |
| Mn 9d SW | -- | -- | -- | -- |
| Mn 9d Mn | -- | -- | -- | -- |
| ***mt8*** |  |  |  |  |
| Mn 2d SW | -- | -- | -- | -- |
| Mn 2d Mn | -- | +2.159± 0.025 | -- | -- |
| Mn 9d SW | -- | -- | -- | -- |
| Mn 9d Mn | -- | -- | -- | +2.04± 0.382 |
| ***abc1b*** |  |  |  |  |
| Mn 2d SW | -- | -- | -- | -- |
| Mn 2d Mn | -- | -- | -- | -- |
| Mn 9d SW | -- | -- | -- | -- |
| Mn 9d Mn | +2.98± 0.334 | -- | -- | -- |
| ***abc4a*** |  |  |  |  |
| Mn 2d SW | -- | -- | -- | -- |
| Mn 2d Mn | +2.55± 0.015 | -- | +2.28± 0.048 | -- |
| Mn 9d SW | +7.91± 0.38 | +6.05± 0.4 | +3.37± 0.312 | +2.17± 0.298 |
| Mn 9d Mn | +6.34± 0.656 | +7.23± 0.253 | +4.35± 0.475 | +3.27± 0.132 |
| ***abc8b*** |  |  |  |  |
| All conditions | -- | -- | -- | -- |
| ***abc1a*** |  |  |  |  |
| Mn 2d SW | -- | -- | -- | -- |
| Mn 2d Mn | +2.16 ± 0.065 | -- | -- | +2.01± 0.123 |
| Mn 9d SW | +4.34± 0.376 | -- | -- | -- |
| Mn 9d Mn | +2.28± 0.155 | +2.13± 0.423 | +2.26± 0.319 | +3.15± 0.222 |
| ***nos*** |  |  |  |  |
| All conditions | -- | -- | -- | -- |
